# Oregon primary care providers as a frontline defense in the War on Melanoma™: improving access to melanoma education

**DOI:** 10.3389/fmed.2025.1427136

**Published:** 2025-03-14

**Authors:** Alyssa L. Becker, Jacob H. Nelson, Alex Verdieck-Devlaeminck, Elizabeth G. Berry, Victoria E. Orfaly, Elizabeth R. Stoos, Jessica Tran, Emile Latour, Vikram N. Sahni, Shuai Xu, Megan Babcock, Anna Bar, Mirna Becevic, Candace Chan, Duncan Chisholm, Kyra Diehl, Karen Edison, Laura K. Ferris, Emilie A. Foltz, Alan C. Geller, Heidi Jacobe, Mariah M. Johnson, Patrick Kinghorn, Justin Leitenberger, Joanna Ludzik, Danielle McClanahan, Stephanie Mengden-Koon, Kelly Nelson, Ryan Petering, Smriti Prasad, Adam Roscher, Stephanie Savory, Emily H. Smith, Susan M. Swetter, Susan Tofte, Martin A. Weinstock, Kevin White, Oliver Wisco, Alexander Witkowski, Sancy A. Leachman

**Affiliations:** 1 Department of Dermatology, Oregon Health and Science University, Portland, OR, United States; 2 John A. Burns School of Medicine, University of Hawai’i at Mānoa, Honolulu, HI, United States; 3 Department of Family Medicine, Oregon Health and Science University, Portland, OR, United States; 4 Biostatistics Shared Resource, Knight Cancer Institute, Oregon Health and Science University, Portland, OR, United States; 5 College of Medicine, Drexel University, Philadelphia, PA, United States; 6 Department of Dermatology, Northwestern University Feinberg School of Medicine, Chicago, IL, United States; 7 Department of Dermatology, University of Missouri School of Medicine, Columbia, MO, United States; 8 Department of Dermatology, University of Pittsburgh Medical Center, Pittsburgh, PA, United States; 9 Elson S. Floyd College of Medicine, Washington State University, Spokane, WA, United States; 10 Department of Social and Behavioral Sciences, Harvard T.H. Chan School of Public Health, Boston, MA, United States; 11 Department of Dermatology, University of Texas Southwestern Medical Center, Dallas, TX, United States; 12 Department of Bioinformatics and Telemedicine, Jagiellonian University Medical College, Kraków, Poland; 13 Department of Dermatology, Stanford University Medical Center and Cancer Institute, Palo Alto, CA, United States; 14 Department of Dermatology, University of Texas MD Anderson Cancer Center, Houston, TX, United States; 15 Department of Dermatology, The Warren Alpert Medical School of Brown University, Providence, RI, United States; 16 Center for Dermatoepidemiology, Providence Veteran Affairs Medical Center, Providence, RI, United States; 17 Dermatology Health Specialists, Bend, OR, United States; 18 Knight Cancer Institute, Oregon Health and Science University, Portland, OR, United States

**Keywords:** melanoma, skin cancer, primary care, family medicine, education, CME, early detection, prevention

## Abstract

Melanoma is one of the deadliest forms of skin cancer but is typically cured with surgical excision when detected early. As an access point to medical care, primary care providers (PCP) play an integral role in early skin cancer detection. However, limited time for examinations and dermatologic training may present barriers to effective skin examination in the primary care setting. As a facet of Oregon Health & Science University’s War on Melanoma™ (WoM), our multi-pronged outreach initiative aims to provide PCPs across Oregon with free, convenient, and effective melanoma education. The WoM PCP education campaign was disseminated starting in May 2019 through primary care networks throughout the state of Oregon to 12,792 PCPs, and education was delivered across several platforms: online multimedia tools, large group didactics, individualized practice-based sessions, and in-person distribution of materials to clinics. To date, 829 PCPs have participated in the online Melanoma Toolkit for Early Detection curriculum, 1,874 providers have attended CME didactics, and 9 clinics have received facilitated meetings by Oregon Rural Practice-based Research Network. Eighty-three clinics (comprising 770 providers) were visited on-site and provided educational materials, and more than 150 PCPs have received a free smartphone dermatoscope to aid in skin examination and e-consultation. OHSU’s WoM has successfully implemented a multifaceted approach to provide accessible melanoma education to PCPs across the state of Oregon. As a result, we hope to encourage appropriate skin examination in the primary care setting and improve PCPs’ diagnostic accuracy and confidence in pigmented lesion evaluation.

## 1 Introduction

Melanoma remains one of the deadliest skin cancers. However, early detection of melanoma can significantly improve survival rates and reduce the need for more aggressive treatment options (1). According to the National Cancer Institute (NCI) Surveillance, Epidemiology and End Results (SEER) data for 2013–2019, the average 5-year survival rate was 99.6% (CI 99.3–99.8%) for patients with localized cutaneous disease at diagnosis, and only 35.1% (CI: 33.8–36.4%) for patients with distant disease at diagnosis (2).

### 1.1 Differential access to dermatologic care and the role of PCPs in melanoma detection

States with a greater density of practicing dermatologists have been shown to be associated with lower mortality to incidence ratios for melanoma (3). However, the distribution of dermatologists in the United States favors urban and coastal regions, leaving rural and underserved areas vulnerable. Additionally, studies based on Cancer Registry data have reported higher incidence and mortality for melanoma in rural areas of the United States (4, 5). In the state of Oregon, there are 110.7 primary care physicians per 100,000 persons, ranking 9th in the United States (6). The ratio of primary care providers (PCP) to dermatologists is even greater when advanced practice practitioners and complementary/alternative medicine providers are classified as PCPs. Based on accessibility, patients are more likely to visit their PCP regularly than a dermatologist. A population-based survey study of 216 melanoma patients showed that 87% of participants had established PCPs while only 20% had a regular dermatologist (7). Thus, PCPs have the opportunity to play an integral role in skin cancer early detection. However, while 63% of participants in the aforementioned study had seen their PCP in the year prior to melanoma diagnosis, most had not received a skin examination (7). Additionally, based on National Health Interview (NHIS) data, only 8% of patients who had seen their PCP in the past year received a skin examination (8). Importantly, a study conducted in Schleswig-Holstein, Germany, the Skin Cancer Research to Provide Evidence for Effectiveness of Screening in Northern Germany (SCREEN) project, showed that PCP training and education in skin cancer detection was associated with a reduction in melanoma mortality (9).

### 1.2 Challenges associated with conducting skin examinations in the primary care setting

Barriers to PCPs implementing skin examinations include limited appointment time to address all patient concerns, inadequate dermatologic education and training, and insufficient data to support routine skin cancer screening by clinicians per the US Preventive Services Task Force (USPSTF) (10–12). Based on electronic health record data, the average primary care visit is 18.0 min (SD = 13.5 min) despite patients often presenting with multiple concerns that may be deemed of higher priority and require extensive counseling (13). This leaves very little time to conduct a full body skin examination, especially when considering the additional time needed for a patient to undress. Even if time was not a factor, many PCPs have limited formal training on skin examination, optimal biopsy methods, or interpretation of dermatopathology reports. The American Academy of Family Physicians (AAFP) includes the performance of skin cancer screening examinations as well as recognition and management of skin cancer in the recommended curriculum guidelines for family medicine residents (14). However, due to the lack of a standardized educational program and universally agreed upon clinical competencies, many residency programs do not provide formal instruction on skin cancer screening and management (10, 15). Only recently has an expert consensus statement been released on proficiency standards for dermoscopy education in primary care (16). Additionally, PCP-oriented skin cancer screening education typically teaches providers to “triage and refer,” but a new educational intervention offering two levels of proficiency “triage and refer” and “diagnose and manage” found that family medicine resident participants demonstrated significant improvement in knowledge and self-efficacy following the training (17). Additionally, it may be unclear to PCPs which patients are appropriate for skin cancer screening. The USPSTF guidelines state that there is insufficient evidence to recommend visual full body skin examination to screen for skin cancer in asymptomatic adolescents and adults; however, this recommendation does not apply to patients with a suspicious skin lesion or those who have elevated risk of skin cancer (11, 12, 18).

### 1.3 Barriers and facilitators in engaging PCPs in continuing medical education

Engaging PCPs in continuing medical education (CME) has unique challenges. Among Hong Kong providers, over 90% of physicians agree that continuous professional development is important in updating knowledge and skills, only 30.7% of non-specialists (compared to 65.4% of specialists) favor continuous professional development to be a requirement for licensure renewal (19). For PCPs, the main barriers to participating in CME non-essential to board licensure include lack of time, perception of work overload, and motivational factors (20). Additionally, dermatologic CME may be deemed less relevant to their daily practice compared to other topics. According to Reis et al., specific to online CME, a lack of digital competence and infrastructure may impede participation. Convenient schedule and location, relevant content, and incentives for participation may improve engagement in CME (19). A survey study conducted in 2018 reported that factors identified as most important in selecting CME activities were topic, quality of content, availability of CME credit, and clinical practice focus (21). Participants in O’Brien Pott et al.’s survey study also reported that they would be most likely to engage in live, online, point-of-care, and print-based CME activities. A meta-analysis aiming to establish the impact of CME interventions on physician knowledge, performance, and patient outcomes, concluded that multifaceted educational programs, longitudinal workshops, interactive small groups, and case discussion interventions delivered to single discipline participant types had the most significant effect sizes (22).

### 1.4 Objective

As a facet of Oregon Health & Science University’s (OHSU) War on Melanoma™ (WoM), our multi-pronged outreach initiative aims to provide PCPs across Oregon access to convenient and effective melanoma education at no cost.

## 2 Methods

The institutional review board at Oregon Health & Science University approved this educational study (STUDY00019372) and waived informed consent for survey participants. Our WoM PCP education campaign was disseminated through the primary care networks of the Oregon Medical Board (OMB), Oregon Medical Association (OMA), Oregon Communication Health Information Network (OCHIN), Oregon Rural Practice-based Research Network (ORPRN), University of Oregon (UO), OHSU’s PCP counsel, Quest Diagnostics, and Castle Biosciences. Education was delivered across several platforms: online multimedia tools,^1^ large group didactics sessions (SAL, EGB, AV), individualized practice-based sessions (SAL, VS), in-person distribution of materials to clinics (Castle Biosciences, VS, and ORPRN), and social media promotions (Quest Diagnostics, University of Oregon). It is important to note that no financial benefits accrued to any group or for-profit company as a part of this distribution. No incentives were offered to increase use of any products offered by these organizations.

### 2.1 Online multimedia tools

WoM hosts a variety of free comprehensive online resources to appeal to different learning styles and preferences. The Melanoma Toolkit for Early Detection (MTED) aims to equip non-dermatology providers with the skills necessary to confidently recognize pigmented skin lesions that are concerning for melanoma (23). The course encompasses a suite of 6 educational modules, featuring recorded discussions conducted by expert dermatologists on the identification of skin cancers. The self-paced modules are expected to take approximately 0.5 h each for a total of 3 h needed for completion. Participants who successfully completed the course were eligible to receive 3 continuing medical education (CME) credits. Additionally, the online resources offer video tutorials on efficient skin examination and biopsy techniques, electronic medical record tools to identify and stratify at-risk patients, billing tools, and unbranded patient education materials (see footnote 1). This innovative “toolkit” design grants participants the flexibility to engage in the complete curriculum or only specific sections most pertinent to their practice and proficiency (SAL, EGB, ERS).

### 2.2 Large group didactics and case-based sessions

Study investigators (SAL, EGB, and AVD) led in-person and virtual CME didactics at statewide PCP meetings. Optional surveys were administered following the CME didactic sessions. Additional study team members (AW and JL) hosted monthly case-based dermoscopy webinars tailored for PCPs.

### 2.3 Individualized practice-based sessions

ORPRN is “a statewide network of primary care clinicians, community partners, and academicians dedicated to studying the delivery of health care, improving the health of Oregonians and reducing rural health disparities.” ORPRN representatives facilitated meetings with primary care providers to discuss their current skin examination practices and provide a tailored introduction to our comprehensive educational resources. The presentations generally lasted 30–60 min and took place in-person or via Zoom. The facilitators provided samples of patient-and staff-facing materials.

### 2.4 Onsite clinic visits and distribution of materials

A study facilitator (VS) conducted onsite visits to primary care clinics across the state to discuss the importance of melanoma screening, distribute educational materials, introduce providers to our comprehensive online multimedia tools, and demonstrate the use of a free smartphone dermatoscope (Sklip, Sklip Inc., Lake Oswego, OR, USA). Clinic sites were selected based on greatest outreach potential, which was defined by high population density, areas containing many primary care practices, and practices with a large number of clinicians. On average, the study facilitator spent 20 min at each clinic site visited. Representatives from Castle Biosciences also distributed educational materials and smartphone dermatoscopes to their PCP network during in-person visits. Additional smartphone dermatoscope attachment devices were also shipped through the United States Postal Service or delivered in-person by various WoM team members and affiliates.

## 3 Results

### 3.1 Large email, newsletter, and digital based communications

Since the program launched in May 2019, 12,792 PCPs were solicited by WoM through targeted email campaigns in collaboration with Oregon healthcare accreditation boards, to participate in the PCP curriculum (Table 1). Over 30,000 digital newsletters and 12,000 printed newsletters were sent out. In addition, an email campaign in collaboration with OCHIN was disseminated to 1,704 PCPs, and the open rate was 21.8% (*n* = 363). Finally, a total of 79,924 impressions (message views) were delivered to healthcare providers through collaboration with the Quest Diagnostic PCP network. See supplementary material for example of messages delivered.

**Table 1:** Credentials of PCPs contacted through the healthcare accreditation boards of Oregon.

Credentials	Number of PCPs contacted (%)
Physician (MD/DO)	4,680 (36.6%)
Physician’s Assistant/Associate (PA)	1,594 (12.5%)
Nurse Practitioner (NP)	3,274 (25.6%)
Chiropractor (DC)	1,960 (15.3%)
Naturopathic Doctor (ND)	1,284 (10.0%)
Total	12,792

### 3.2 Online multimedia tools

Across the state, 829 PCPs have participated in the MTED curriculum. From 2019 to 2022, primary care-related content on OHSU’s WoM website has been viewed a total of 9,951 times by 7,450 unique users (Table 2).

**Table 2:** Website analytics report for the War on Melanoma PCP toolkit landing page stratified by year.

Year	Page Views	Unique users	Avg time on page (min:sec)	Avg engagement rate^*^
2019	2,909	2,099	05:25	81.1%
2020	2,779	1,981	06:06	81.0%
2021	1,525	1,165	03:35	76%
2022	2,738	2,205	05:06	92.1%
Overall	9,951	7,450	05:18	82.5%

* Engagement rate is defined by the number of users who click on a link within the webpage.

### 3.3 Large group didactics and case-based sessions

Over 10 CME lectures were led by melanoma experts across the state with a total of 1,874 PCP attendees. The post-lecture survey results are detailed in Table 3. Statements were rated on a scale of 1–5 with 1 = strongly disagree, and 5 = strongly agree. On average, attendees who completed our optional post-lecture survey agreed (mean response >3 on a 5-point scale) that the presentations were relevant to their practice, will influence their clinical practice, and that content was conveyed effectively. In-person large group CME didactics provided by melanoma experts offered a deeper dive into melanoma detection, but participants identified key areas that could be improved. Many post-survey respondents voiced the need for additional clinical and dermoscopic images of melanoma to hone their triage and diagnostic skills. Comments also mentioned a lack of interest in detailed information regarding melanoma management and the desire for additional practical tips for PCPs.

**Table 3:** CME didactics post-survey results.

Statement	Average rating (scale of 1-5^a^)	Number of responses
This presentation was relevant to my practice	3.58	615
I will make changes in patient care based on the information presented	3.41	575
The content of the presentation was conveyed effectively	3.50	610

a 1 = strongly disagree, 2 = somewhat disagree, 3 = neutral, 4 = somewhat agree, 5 = strongly agree.

### 3.4 Individualized practice-based sessions

Of the 61 clinics the ORPRN team attempted to contact, 9 clinics (15%) opted to host a practice facilitator for a tailored introduction to MTED with approximately 69 participants. Four of the participating clinics were located in frontier locations, 2 in rural locations, 1 in a rural/urban location, and 2 in an urban location. Five clinics opted to receive educational materials (1 frontier, 3 rural, and 1 rural/urban). Eight clinics declined any engagement. Thirty-six clinics failed to respond to ORPRN regarding WoM’s PCP education initiative.

### 3.5 Onsite clinic visits and distribution of materials

From May 2022 to June 2022, 83 clinics were visited onsite in (number of cities and number of counties) and provided educational materials, impacting 770 providers (Table 4). More than 150 PCPs have received free smartphone dermatoscopes to date, with user instructions and resources for triage with a dermoscopy expert at our institution.

**Table 4:** Locations of clinics visited onsite^a^.

City	Population in 2022^b^	Proportion of Oregon’s total population^c^, %	Clinics visited, *n* (%)
Salem, OR	179,605	4.2%	11 (13.3%)
Medford, OR	88,357	2.1%	7 (8.4%)
Corvallis, OR	59,434	1.4%	4 (4.8%)
Grants Pass, OR	39,993	0.9%	6 (7.2%)
McMinnville, OR	34,515	0.8%	7 (8.4%)
Newberg, OR	25,767	0.6%	4 (4.8%)
Klamath Falls, OR	22,501	0.5%	9 (10.8%)
Ashland, OR	21,642	0.5%	7 (8.4%)
Hermiston, OR	19,973	0.5%	5 (6.0%)
Pendleton, OR	16,894	0.4%	6 (7.2%)
La Grande/Elgin, OR	15,182	0.4%	7 (8.4%)
Ontario, OR	11,845	0.3%	7 (8.4%)
Baker City, OR	10,178	0.2%	3 (3.6%)
Total	545,886	12.7%	83 (100.0%)

a Sites visited by VS.

b Population data reported by Portland State University’s Population Research Center.

c The certified estimate of Oregon’s population in 2022 was 4,281,851.

## 4 Discussion

Beginning in May 2019, OHSU’s WoM implemented a broad, multifaceted, education-based outreach program to PCPs across the state of Oregon. The program consists of online multimedia tools, large group didactics, individualized practice-based sessions, and on-site clinic visits, to offer free, accessible melanoma educational programming. The outreach was accomplished through collaboration with Oregon Medical Board (OMB), Oregon Medical Association (OMA), Oregon Communication Health Information Network (OCHIN), Oregon Rural Practice-based Research Network (ORPRN), OHSU’s PCP counsel, and with two industry collaborators. ORPRN is a provider network dedicated to improving the health of rural Oregonians through education and research (24). OCHIN, a health information network, shares a similar goal of health equity through innovative solutions (25). Both of these networks are involved in research and well-funded by state and federal sources.

**Figure 1:**
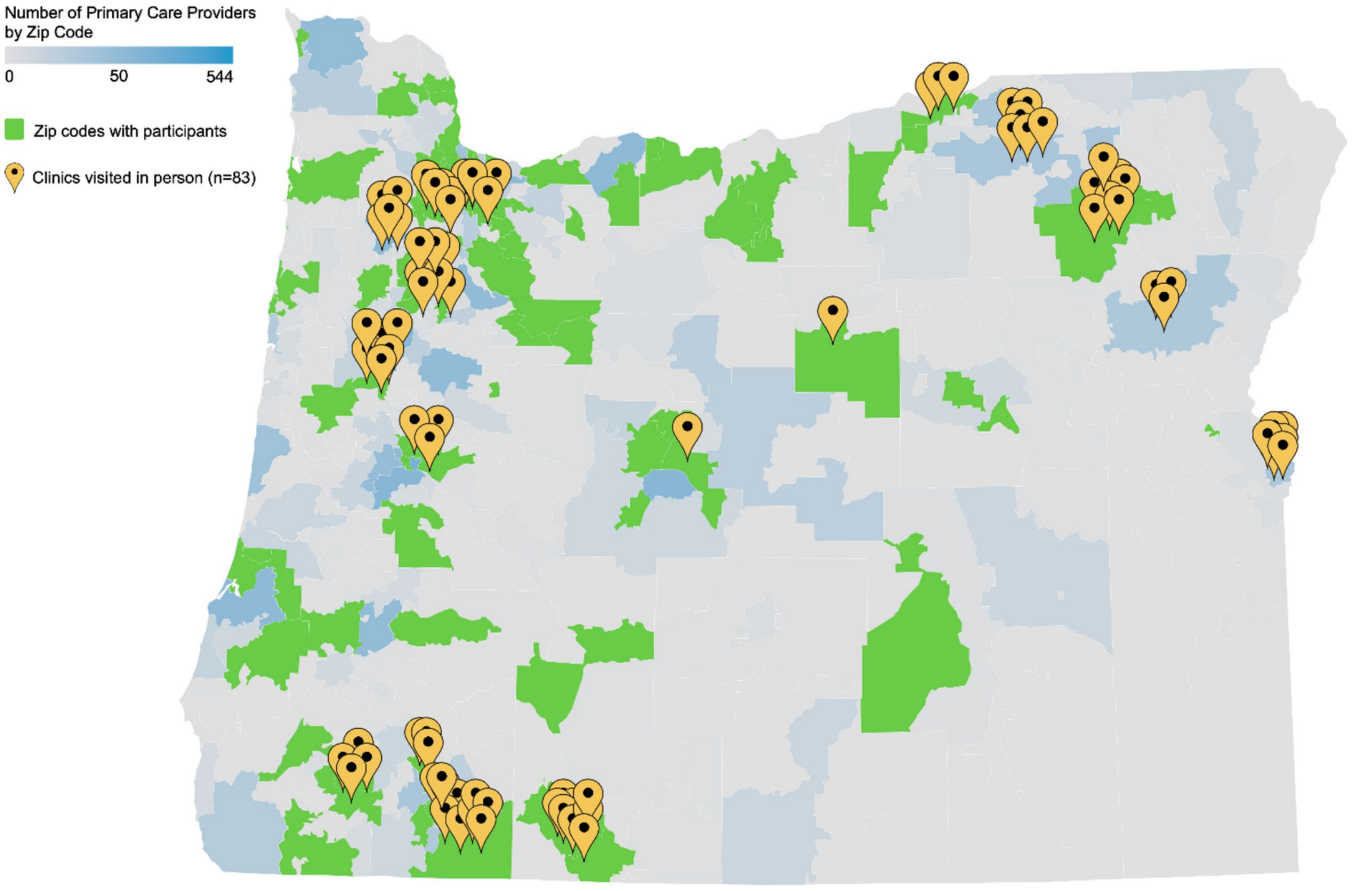
Primary care outreach density/distribution.

Our education initiative achieved good geographic distribution across the state (Figure 1), and the variety of tools available in the educational toolkit permitted learners to self-select the learning methods that are best suited to their practice, schedule, and learning style.

Map of the State of Oregon, with density of primary care providers (PCP) by practice zip code. Gray indicates there are zero PCP practice addresses listed in each zip code. Gray-blue gradient indicates the number of PCP with practice addresses listed in each zip code. Green shading indicates that a given zip code contains greater than zero PCPs participating in the curriculum. Yellow map markers indicate locations of clinics or hospitals that received in-person presentations and invitations to participate in the curriculum (Map created with Datawrapper).

### 4.1 Online multimedia tools

Providing meaningful education in a time efficient manner is crucial to engaging busy healthcare providers, and the flexible “toolkit” design of our web-based resources allowed participants to engage in the content most relevant to their pre-existing knowledge base.^2^ A previous study confirmed that healthcare providers were highly likely to engage in online CME because learning could be done when clinicians had time and at their own pace (21).

The 2019 MTED pilot study demonstrated a promising 6 percentage point average improvement in identifying benign and malignant lesions (95% CI: 3.5 to 8.6, *p* < 0.001 paired t-test; averages of 82.9% on the pretest to 89.0% on the post-test), accompanied by a 44.2% improvement in diagnostic confidence (95% CI 29.3 to 59.0%, *p* < 0.001, McNemar’s test) following completion of the online training modules (23). A larger sample size of participants who completed both pre-and post-surveys is required for additional quantification of the online training’s impact on PCP triage accuracy. Additional longitudinal assessment of in-clinic behavior changes would also be helpful in assessing the full impact of our online resources.

One limitation of the online education platform that we utilized was the lack of available user engagement analytics software. Analytics software would have allowed us to determine which topics, if any, were the most utilized. This would have also potentially provided participant demographic data, thereby allowing us to identify groups who were not effectively reached that may benefit from additional outreach and education.

### 4.2 Large group didactics and case-based sessions

Healthcare providers who participated in the INFORMED curriculum expressed a need for more detailed skin cancer detection instruction and assistance with challenging patient cases (26). These challenges of online education can be addressed through live instruction with pigmented skin lesion experts. A 2018 survey of 500 healthcare providers revealed a preference for live CME, mainly because they felt topics were best taught using this modality (21). However, it should be noted that the effectiveness of lectures may be limited to auditory learners (27). An alternative method, case-based learning, which links theory and practice, has reportedly been preferred by 84% of medical students over traditional lectures, and has shown improvements in motivation, satisfaction, and engagement (28, 29). It is unknown whether these data discrepancies are related to generational preferences. Regardless, live large group didactics and dermoscopy webinars may serve as a beneficial supplement to online educational methods or previous knowledge.

Monthly live case-based, discussion-oriented dermoscopy webinars tailored for PCP audiences allowed for spaced repetition and also a safe space to ask pigmented lesion experts questions about challenging cases encountered during patient care in the real world. These webinars were scheduled during the noon lunch hour on Fridays to maximize attendance, which resulted in an estimated 75 providers participating throughout the course.

One important learning point was recognizing the importance of having a diverse selection of modalities for education. Some providers preferred in-person training experiences, while some only participated in online options. While in-person training may be preferred, it is limited by its resource-intensive nature. Future efforts will involve consideration of achieving a finer balance in allocating resources to increase access to in-person training modalities.

### 4.3 Individualized practice-based sessions

The need to improve access to melanoma care in rural areas was highlighted in a study demonstrating that patients in rural zip codes had higher melanoma prevalence and travelled much greater distances for treatment compared to patients residing in urban areas (30). ORPRN’s purpose is to address these disparities, and it is one of the most successful programs of its type in the United States. Their mission is to improve health outcomes and equity for persons across the state of Oregon (24). ORPRN’s outreach efforts for the WoM project concentrated on PCPs in rural and frontier regions of Oregon due to the scarcity of dermatologists in these communities. While only 9 out of 61 clinics that we contacted engaged in a practice-based session, this engagement rate is similar or outperforms other initiatives led by ORPRN per their representatives. Although this is typical, we need to find strategies to increase participation (opportunities for follow-up etc).

Reasons cited for declining a practice-based session included staffing shortages, impending EMR changes, and limited capacity to engage in additional quality improvement work. Limited time availability is the common theme across these declinations, making it challenging to overcome. It may be possible to improve participation with increased incentive if resources are available. Even with personalized outreach, these results highlight the barriers faced by rural and frontier healthcare providers in engaging in CME. Contacted clinics acknowledged the importance of melanoma education, and no clinics indicated that they had been approached to engage in melanoma education previously. Other underlying barriers that are difficult to address are negative attitudes in individuals we are attempting to reach, and potential “burn out” from high stress environments. While the participation rate was less than optimal, we now further understand barriers to participation and will implement strategies to overcome these in future outreach efforts.

### 4.4 Onsite clinic visits and distribution of materials

The primary aim of employing “door-to-door” canvassing as one of our outreach methods was to reach clinics and providers that may otherwise miss or ignore other types of communication. In 2022, Litmus found that people spend just 9 s, on average, looking at an email (31). However, our team members report spending an average of 20 min at each clinic site visited reviewing educational resources. A study regarding door-to-door surveys concluded that this method is valuable in certain research contexts, especially when spending time in a community, conducting observations, and building relationships are central to the overarching goal (32). It should be noted that the success of this method of outreach is highly dependent on individual interpersonal skills and expertise in the topic being shared (33).

In addition to providing information about our educational resources, onsite clinic visits also included a demonstration of a smartphone dermatoscope that PCPs could obtain for free. Dermoscopy improves the diagnostic accuracy for melanoma compared to “naked-eye” visualization alone; however, the high cost of dermatoscopes limits their use by non-dermatology providers (34–37). Furthermore, the diagnostic accuracy from the use of dermoscopy is highly user-dependent, as additional intensive training for pattern recognition of features and routine practice using this technique is required for proficiency. By providing smartphone dermatoscopes to PCPs at no cost as well as in-person training, we attempted to improve their skin examination capabilities and equip them with a tool to quickly capture high-resolution images of skin lesions for inclusion in EMR documentation and e-consultations with dermatologists.

### 4.5 Conclusion and future directions

OHSU’s WoM has launched a robust melanoma education program that is accessible to PCPs across the state of Oregon (38). Individual components of the program were evaluated for integration into the community. While it was not possible to cross-compare different aspects of our program to identify the most effective means of increasing PCP melanoma early detection, long-term impact of the education effort will be assessed through cancer registry data and all-payer all-claims databases. As part of the WoM campaign, we have coupled the outreach to PCPs with a statewide public education campaign encouraging the general population to check their own skin and direct any concerns to their provider for evaluation (38, 39). We hypothesize that a coupled approach will maximize melanoma early detection in Oregon and that this can be translated to other states. Future investigations will also implement new strategies to reach PCPs throughout Oregon. This strategy will effectively deliver education and resources and increase their ability to detect melanoma before it becomes highly morbid or lethal. These data will provide valuable insights into the role of PCPs in the early detection of melanoma and the impact of the WoM program.

## Data Availability

The original contributions presented in the study are included in the article/Supplementary materials, further inquiries can be directed to the corresponding author.

## Footnotes

1
https://www.ohsu.edu/war-on-melanoma/melanoma-early-detection-toolkit

2
https://www.ohsu.edu/war-on-melanoma/melanoma-early-detection-training
