# Correction: Oregon primary care providers as a frontline defense in the War on Melanoma™: improving access to melanoma education

**DOI:** 10.3389/fmed.2026.1827805

**Published:** 2026-03-24

**Authors:** Alyssa L. Becker, Jacob H. Nelson, Alex Verdieck-Devlaeminck, Elizabeth G. Berry, Victoria E. Orfaly, Elizabeth R. Stoos, Jessica Tran, Emile Latour, Vikram N. Sahni, Shuai Xu, Megan Babcock, Anna Bar, Mirna Becevic, Candace Chan, Duncan Chisholm, Kyra Diehl, Karen Edison, Laura K. Ferris, Emilie A. Foltz, Alan C. Geller, Heidi Jacobe, Mariah M. Johnson, Patrick Kinghorn, Justin Leitenberger, Joanna Ludzik, Danielle McClanahan, Stephanie Mengden-Koon, Kelly Nelson, Ryan Petering, Smriti Prasad, Adam Roscher, Stephanie Savory, Emily H. Smith, Susan M. Swetter, Susan Tofte, Martin A. Weinstock, Kevin White, Oliver Wisco, Alexander Witkowski, Sancy A. Leachman

**Affiliations:** 1 Department of Dermatology, Oregon Health and Science University, Portland, OR, United States; 2 John A. Burns School of Medicine, University of Hawai'i at Mānoa, Honolulu, HI, United States; 3 Department of Family Medicine, Oregon Health and Science University, Portland, OR, United States; 4 Biostatistics Shared Resource, Knight Cancer Institute, Oregon Health and Science University, Portland, OR, United States; 5 College of Medicine, Drexel University, Philadelphia, PA, United States; 6 Department of Dermatology, Northwestern University Feinberg School of Medicine, Chicago, IL, United States; 7 Department of Dermatology, University of Missouri School of Medicine, Columbia, MO, United States; 8 Department of Dermatology, University of Pittsburgh Medical Center, Pittsburgh, PA, United States; 9 Elson S. Floyd College of Medicine, Washington State University, Spokane, WA, United States; 10 Department of Social and Behavioral Sciences, Harvard T.H. Chan School of Public Health, Boston, MA, United States; 11 Department of Dermatology, University of Texas Southwestern Medical Center, Dallas, TX, United States; 12 Department of Bioinformatics and Telemedicine, Jagiellonian University Medical College, Kraków, Poland; 13 Department of Dermatology, Stanford University Medical Center and Cancer Institute, Palo Alto, CA, United States; 14 Department of Dermatology, University of Texas MD Anderson Cancer Center, Houston, TX, United States; 15 Department of Dermatology, The Warren Alpert Medical School of Brown University, Providence, RI, United States; 16 Center for Dermatoepidemiology, Providence Veteran Affairs Medical Center, Providence, RI, United States; 17 Dermatology Health Specialists, Bend, OR, United States; 18 Knight Cancer Institute, Oregon Health and Science University, Portland, OR, United States

**Keywords:** melanoma, skin cancer, primary care, family medicine, education, CME, early detection, prevention

The affiliation “Department of Bioinformatics and Telemedicine, Jagiellonian University Medical College, Kraków, Poland” was omitted for author Joanna Ludzik. Joanna Ludzik should have “Department of Dermatology, Oregon Health and Science University, Portland, OR, United States” and “Department of Bioinformatics and Telemedicine, Jagiellonian University Medical College, Kraków, Poland”.

The original version of this article has been updated.

