# Supplementary material for: Oregon primary care providers as a frontline defense in the War on Melanoma™: improving access to melanoma education
Source: Front Med (Lausanne). 2025 Mar 14;12:1427136. doi: 10.3389/fmed.2025.1427136 (PMC11949923; doi:10.3389/fmed.2025.1427136)
Supplement: Supplementary file 1 [file Data_Sheet_1.pdf]

OHSU

What you need to know about:

Preventing skin cancer in children

Sun protection

Protecting your child from the sun is one of the best ways to prevent skin cancer. UV rays from the sun, which cause skin cancer, are strongest from 10 a.m. – 3 p.m., so it is important to be especially careful during those times. Tanning beds are a source of harmful UV rays and are never safe to use.

When in the sun, please have your child

- Wear clothes that protect their skin from sunlight (look for the UPF tag. Brands include Columbia, Coolibar, and Sunday Afternoons)
- Wear a wide-brimmed hat that shades their face and ears
- Wear sunglasses
- Stand in the shade whenever possible
- Apply sunscreen every two hours while out in the sun
    - Re-apply more often if you are sweating or swimming
    - Sunscreen alone is not enough to protect you from skin cancer; it should be used *in addition* to the other sun safety tips

What sunscreen should I use for my child?

- Children over six months old should wear sunscreen. Children under six months old can use sunscreen on small areas if there is no other way to protect the skin. For infants, the only active ingredients should be zinc oxide and/or titanium dioxide.
- Find a sunscreen with an **SPF of at least 30** and a label that says "**broad-spectrum**" protection.
- **Ingredients are important**. The best sunscreens use minerals, such as zinc oxide or titanium dioxide, instead of chemical ingredients to block the sun. They are considered safe by the

FDA, even for children. They are also better for the environment and will not damage coral reefs.

- Sunscreens that use chemicals are okay, but not ideal; we do not know the long-term safety of chemical-based sunscreens.
- Recommended brands:
    - Aveeno Natural Protection
    - Aveeno Positively Mineral
    - Banana Boat “Simply Protect Kids SPF 50+
    - Blue Lizard Baby
    - Badger Active
    - Banana Boat Baby
    - CoTz
    - Elta MD UV Pure Broad-Spectrum
    - Trader Joe’s Mineral
    - Neutrogena Pure & Free Baby
    - Neutrogena Sensitive Skin
    - Neutrogena
    - Thinksport
    - Vanicream (original)
    - Spray options: Sun bum Mineral Snscreen Spray, EltaMD UV Aero, Countersun Mineral Sunscreen Mist
    - Chemical options: Neutrogena “Dry Touch,” Neutrogena Hydro Boost Water Gel Sunscreen, Elta MD UV Clear, Banana Boat Simply Protect Spray, Cerave Sunscreen Face Lotion.
- Remember: Any sunscreen is better than no sunscreen at all.
- Please see the Society for Pediatric Dermatology's Sun Protection handout for additional information: bit.ly/2Yv5hnC

Moles and spots

When should I worry about my child's moles?

- It is normal for moles to grow as your child grows.
- Expect that new, normal, moles might appear over your child's lifetime up until age 35.
- Watch their current moles for unusual changes.
- Watch your child's moles and let your child's health care provider know right away if you notice a mole that does not look like the others or seems to be changing quickly.

Free OHSU MoleMapper App (available on your iPhone or iPad)

Change or growth of moles is one of the strongest signs of skin cancer. With the MoleMapper app, you can use your iPhone or iPad to photograph and track your moles each month. The MoleMapper can help you and your health care provider see if your moles are changing in a concerning way over time.

To download: go to the App Store, search for "MoleMapper Melanoma Study," and tap "GET."
