# Supplementary material for: Oregon primary care providers as a frontline defense in the War on Melanoma™: improving access to melanoma education
Source: Front Med (Lausanne). 2025 Mar 14;12:1427136. doi: 10.3389/fmed.2025.1427136 (PMC11949923; doi:10.3389/fmed.2025.1427136)
Supplement: Supplementary file 2 [file Data_Sheet_2.pdf]

OHSU

What you need to know about:

Melanoma and other skin cancers

Melanoma

Melanoma is the most deadly form of skin cancer. When found early, it is almost always curable, which is why it is important to check your skin and talk to your health care provider if anything looks different.

To catch melanoma

- Check your skin every month for new or changing moles or spots.
- Let your medical provider know if you see any of the warning signs of melanoma or other skin cancers.

What does melanoma look like?

Look for new moles (spots on your skin) or moles that are changing in size, shape or color. As you get older, your moles may slowly change, but a mole should never change quickly (within a few weeks or months).

Melanoma can happen anywhere – not just areas exposed to the sun. When you are checking your skin, be sure to look at your whole body.

Melanoma warning signs

A new mole (especially if you are 55 or older) or a changing mole.	One part of the mole that is growing differently from the rest of the mole. Look for difference in color, shape or size.	A mole that is different from your other moles
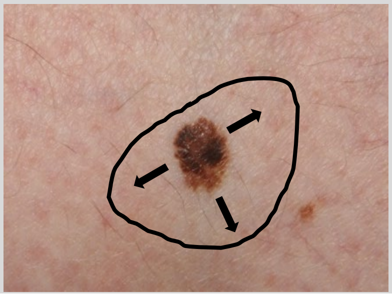	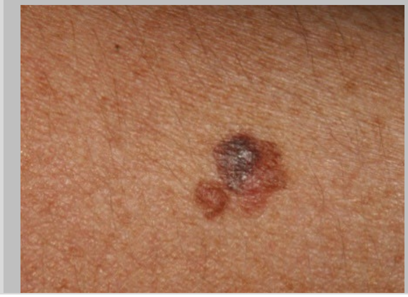	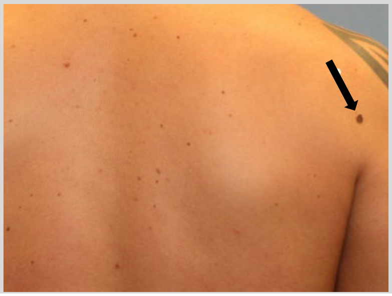

What does melanoma look like?

Below are a few examples of how melanoma might look.

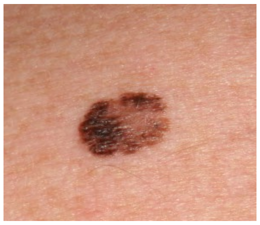

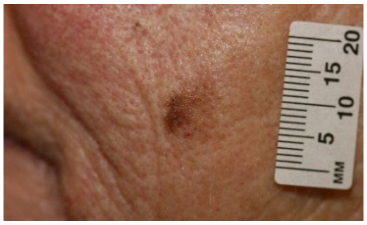

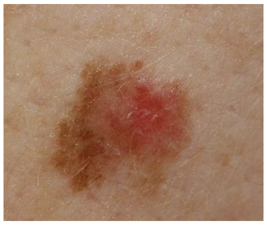

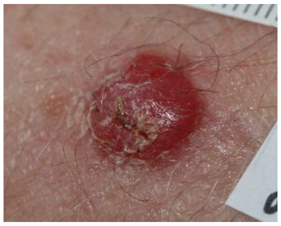

Note that just because a mole is different, does not mean it is melanoma. You should always talk to your health care provider if you see any melanoma warning signs.

Citation: Mole images: 'SkinCancer909: a textbook of skin cancer for medical students' by Jonathan Rees. www.skincancer909.com

Other types of skin cancer

Not all skin cancers are melanoma, although melanoma is the most dangerous.

The most common other types of skin cancer include:

- Squamous cell carcinoma
- Basal cell carcinoma

These skin cancers are usually found on parts of the body that get a lot of sunlight, including your:

- Face
- Ears
- Top of the head
- Neck
- Arms

Squamous Cell Carcinoma (SCC)

Squamous cell carcinomas are cancerous growths that look like scaly or crusty lumps or sores.

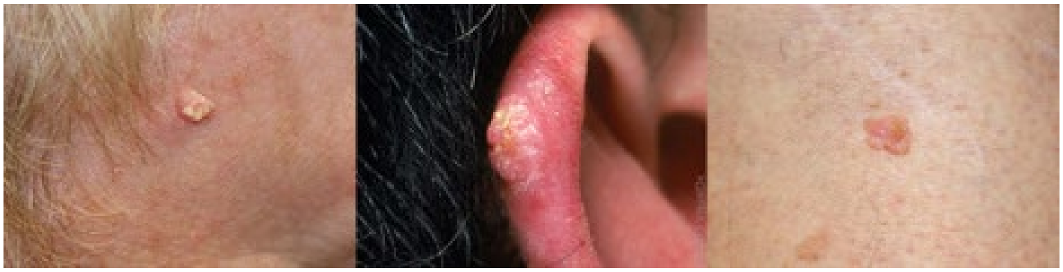

Actinic Keratosis (AK)

An actinic keratosis (AK) is a rough, scaly patch caused by sun damage. Although AKs are not cancer, they are precancerous and can become squamous cell carcinoma if they are not treated.

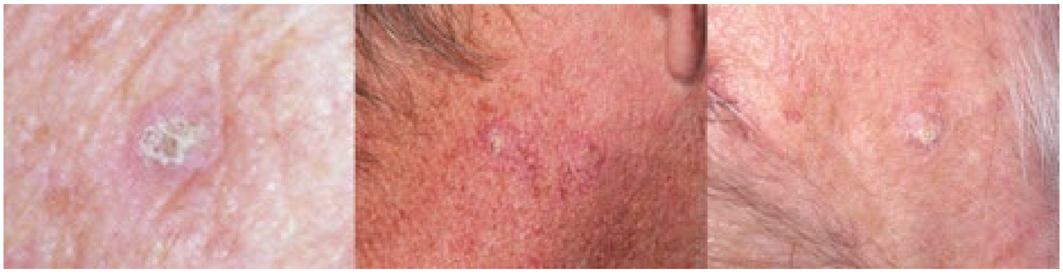

Basal Cell Carcinoma (BCC)

Basal cell carcinomas are the most common form of skin cancer. BCCs often have edges that bleed and scab. They might also look like a black, blue, brown, or a slightly scaly spot that grows over time.

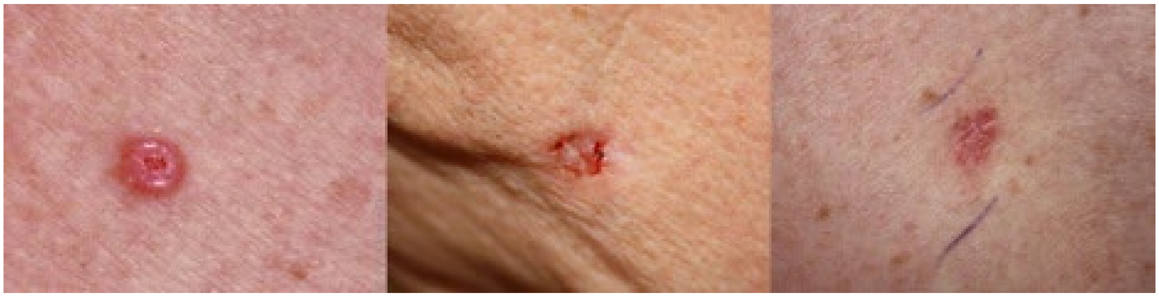

Preventing skin cancer

Sun protection

Protecting yourself from the sun is one of the best ways to prevent skin cancer. UVB rays from the sun, which cause skin cancer, are strongest from 10 am – 3 pm, so it is important to be especially careful during those times. Whenever you are in the sun:

- Wear clothes that protect your skin from sunlight (look for the UPF tag)
- Wear a wide-brimmed hat that shades your face and ears
- Wear sunglasses
- Stand in the shade whenever possible
- Apply sunscreen every two hours while out in the sun

    - Re-apply more often if you are sweating or swimming
    - Sunscreen alone is not enough to protect you from skin cancer; it should be used *in addition* to the other sun safety tips.

What sunscreen should I use?

- Find a sunscreen with an SPF of at least 30 and a label that says "broad-spectrum" protection.
- **Ingredients are important**. The best sunscreens use minerals, such as zinc oxide or titanium dioxide, instead of chemical ingredients to block the sun. They are considered safe by the FDA, even for children.
- Sunscreens that use chemicals are okay, but not ideal; we do not know the long-term safety of chemical-based sunscreens.
- Recommended brands:
    - Blue Lizard Baby
    - Banana Boat Baby
    - Trader Joe's Mineral
    - Neutrogena Pure & Free Baby
    - CoTz
    - Elta MD UV Pure Broad-Spectrum
- Remember: Any sunscreen is better than no sunscreen at all.

Self-exams

Most skin cancer is first seen by you or your partner rather than your doctor. To do a good skin check:

- Look at your whole body, including your fingernails and between your toes.
- Look at each part of your body in the same order every time to make sure you don't miss any spots.
- Use a mirror to look at the top or back of your head, back, under your breasts, genitals or buttocks.
- If you have a partner, they can help check hard-to-see areas.
- Let your health care provider know about any new or changing spots.

Free OHSU MoleMapper App (available on your iPhone or iPad)

Change or growth of moles is one of the strongest signs of skin cancer. With the MoleMapper app, you can use your iPhone or iPad to photograph and track your moles each month. The MoleMapper can help you and your health care provider see if your moles are changing in a concerning way over time.

To download: go to the App Store, search for "MoleMapper Melanoma Study," and tap "GET."

More information about preventing, finding and treating skin cancer

Visit www.StartSeeingMelanoma.com or www.WarOnMelanoma.org.

What does the "War on Melanoma™" mean?

The War on Melanoma™ is an OHSU research program designed to stop death due to melanoma through early detection. If you have any questions about the program, you can email WarOnMelanoma@ohsu.edu, call 844-300-SPOT (7768), or visit www.WarOnMelanoma.org.

Interested in research opportunities? Stay up-to-date by joining the Melanoma Community Registry: www.ohsu.edu/war-on-melanoma/melanoma-community-registry.

Questions?

If you have any questions about the app, you can contact WarOnMelanoma@ohsu.edu or call 844-300-SPOT (7768).
