# Supplementary material for: Oregon primary care providers as a frontline defense in the War on Melanoma™: improving access to melanoma education
Source: Front Med (Lausanne). 2025 Mar 14;12:1427136. doi: 10.3389/fmed.2025.1427136 (PMC11949923; doi:10.3389/fmed.2025.1427136)
Supplement: Supplementary file 3 [file Data_Sheet_3.pdf]

Using Epic tools for Melanoma Clinic

OHSU DERMATOLOGY

Language

SmartPhrase (aka DotPhrase) – custom text template used while charting.

Annotated as .[name]

SmartList – drop-down menus that can be customized and inserted into SmartPhrases to force the charting individual to select from a list of pre-determined responses.

Annotated as {[name]}

SmartLink – automatically pulls information from patient chart (i.e. age, family hx, diagnosis, etc...)

Annotated as @[name]@

Wildcard – forces free-text response by charting individual in a SmartPhrase. Note that clicking the ‘F2’ key will take you to the next (***) wildcard.

Annotated as ***

SmartPhrases

Name	Purpose
.MELRISK	Melanoma risk assessment
.SKINCANCERAVS	Skin cancer education after-visit summary handout
.MELHPI	Melanoma history of present illness
.MELPE	Melanoma physical examination
.MELROS	Melanoma review of systems
.SKINCANCERPE	Skin cancer physical examination
.SKINCANCERPUNCHBX	Skin cancer single punch biopsy
.SKINCANCERPUNCHBXS	Skin cancer multiple punch biopsies
.SKINCANCERSHAVEBX	Skin cancer single shave biopsy
.SKINCANCERSHAVEBXS	Skin cancer multiple shave biopsies
.MOLEMAPPER	OHSU MoleMapper information & download instructions

.MELRISK

Series of questions to ask/observe of the patient. Includes images for reference

Questions are listed in **bold** with possible answers annotated as {SmartLists}. Each response is associated with a (risk score point value)

After completing the assessment, manually add all (points) together for a total score

Recommendations for education and medical provider exams are included (per risk level) at the end. Ultimately, physician discretion will determine how to proceed moving forward, but this should provide some framework and questions to consider based off the patient’s level of risk.

.MELRISK

Step 1)

Type '.MELRISK' while charting and hit the enter key

Step 2)

Go through questionnaire (see later slides) and answer each **question.** Every {answer} will have an associated point value

Step 3)

Assign a risk score by adding the total points together

Step 4)

Use the recommendations chart and the associated risk level to aid in education and treatment/management decisions

What is the patient's current eye color?

SmartList	Answer	Points
{MelR eye color:20185}	Blue<br>Green<br>Hazel<br>Brown<br>Dark brown/black	3 points<br>3 points<br>3 points<br>0 points<br>0 points

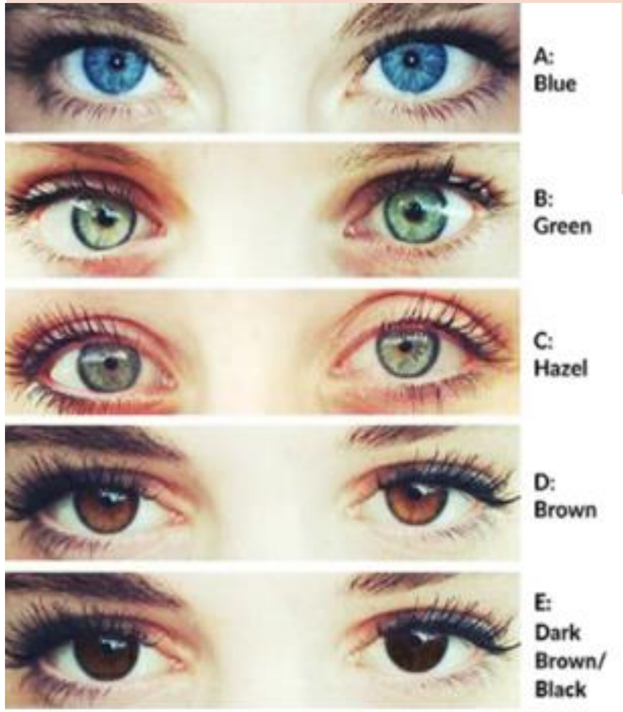

What was the patient’s natural hair color at 18?

SmartList	Answer	Points
{MelR hair color:20186}	Blonde	1 point
	Strawberry blonde	3 points
	Red	3 points
	Auburn	3 points
	Light brown	1 point
	Dark brown	0 points
	Black	0 points

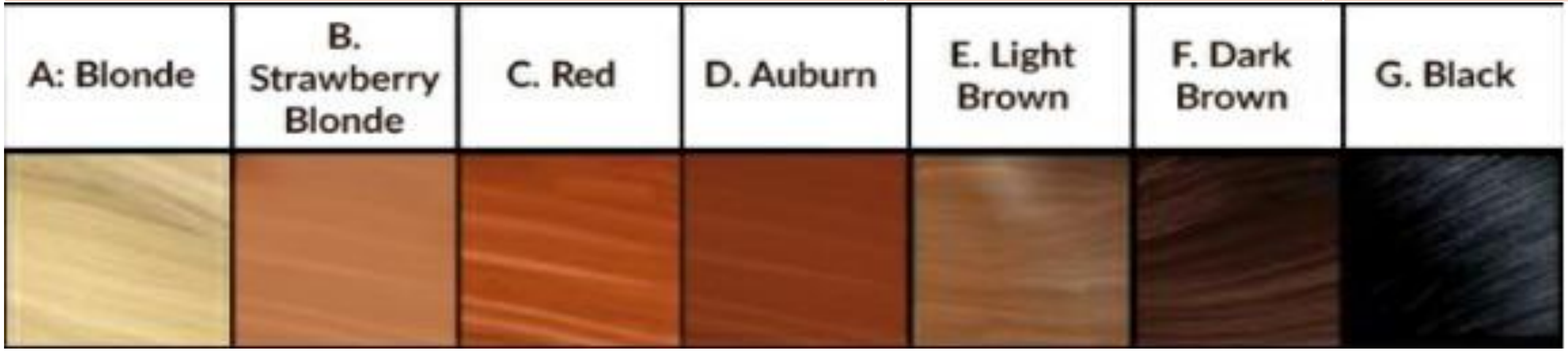

What is the patient’s Fitzpatrick skin type?

SmartList	Answer	Points
{MelR Fitzpatrick skin type:20183}	Skin type I<br>Skin type II<br>Skin type III<br>Skin type IV<br>Skin type V<br>Skin type VI	1 point<br>1 point<br>1 point<br>0 points<br>0 points<br>0 points

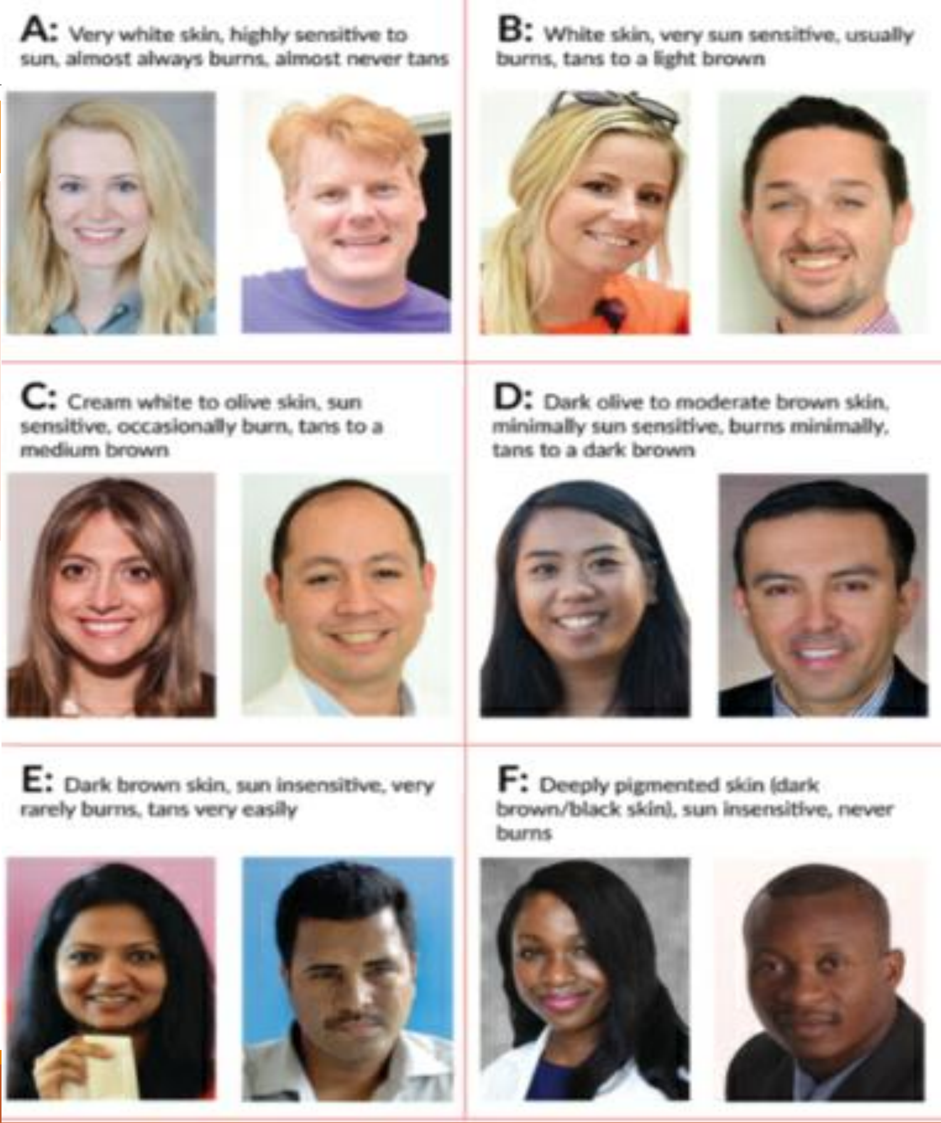

As a child, how many freckles did the patient have?

SmartList	Answer	Points
{MelR freckles:20187}	None<br>Few<br>Many	0 points<br>1 point<br>1 point

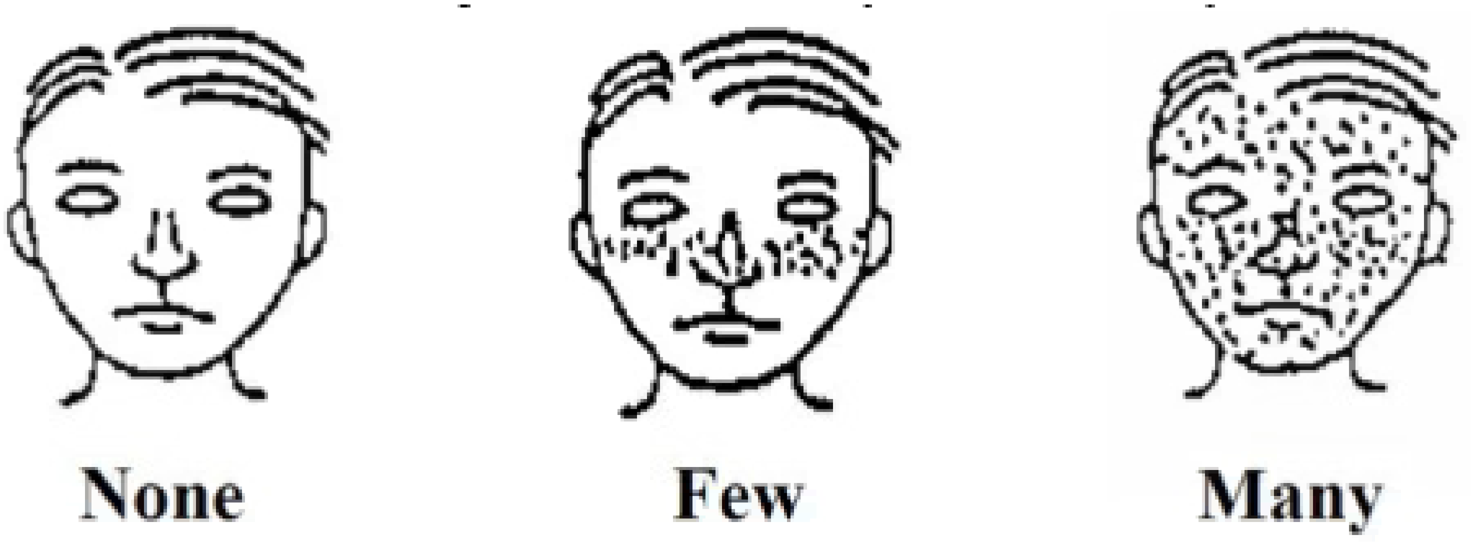

Estimate the number of moles on the patient’s body

SmartList	Answer	Points
{MelR moles:20172}	Less than 15<br>16-60<br>61-80<br>>80	0 points<br>1 point<br>3 points<br>9 points

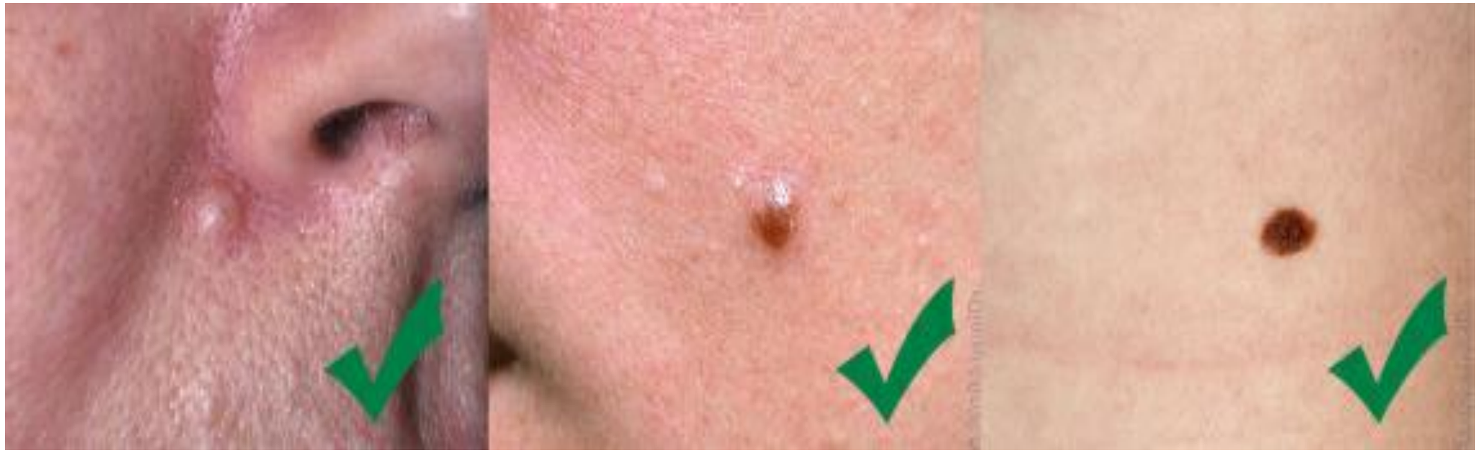

Has a health care provider ever diagnosed the patient with an actinic keratosis (usually treated with freezing or prescription cream)?

SmartList	Answer	Points
{MelR actinic keratosis: 20182}	Yes<br>No<br>Patient does not recall	3 points<br>0 points<br>0 points

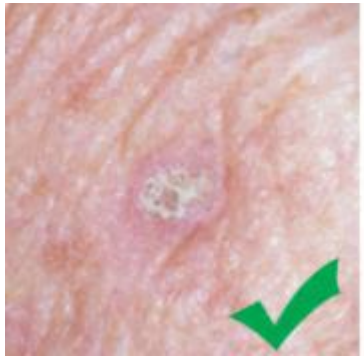
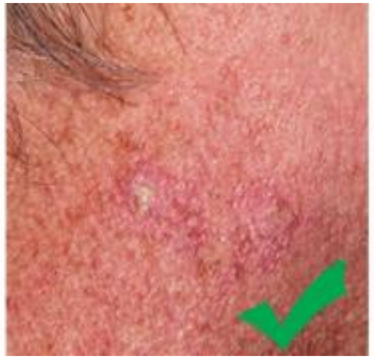
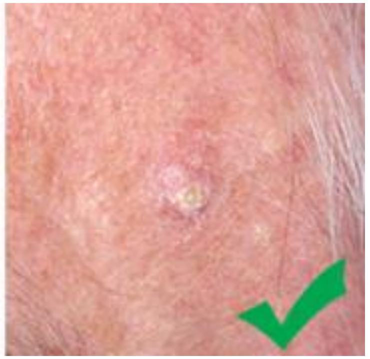

Please indicate which statement is most accurate regarding patient gender and indoor tanning practice

SmartList	Answer	Points
{MelR tanning:20184}	Patient is a male who has used indoor tanning<br>Patient is a male who has never used indoor tanning<br>Patient is female and started tanning before age 30<br>Patient is female and started tanning between the ages of 30 and 39<br>Patient is female and started tanning after the age of 40<br>Patient is female and does not remember when they started indoor tanning<br>Patient is female and has never used indoor tanning	1 point<br>0 points<br>9 points<br>3 points<br>1 point<br>1 point<br>0 points

Has the patient ever had a severe sunburn (blisters or symptoms that lasted >2 days)?

SmartList	Answer	Points
{MelR sunburn:20327}	Yes<br>No	1 point<br>0 points

Has the patient ever been diagnosed with melanoma?

SmartList	Answer	Points
{MelR melanoma:20328}	Yes<br>No	9 points<br>0 points

Have any first-degree family members of the patient (parents, siblings, children) ever been diagnosed with melanoma?

SmartList	Answer	Points
{MelR family hx melanoma:20328}	Yes<br>No	3 points<br>0 points

Has the patient had an organ transplant or received long courses (>1 month) of immunosuppressive medications for other medical conditions?

SmartList	Answer	Points
{MelR melanoma:20328}	Yes<br>No	9 points<br>0 points

To calculate the Melanoma Risk Score, please add all the points together from the section above

RECOMMENDATIONS STRATIFIED BY MELANOMA RISK LEVEL				
	**Low Risk (Total: 0 points)**	**Some Risk (Total: 1-3 points)**	**Moderate Risk (Total: 4-8 points)**	**High Risk (Total: 9 or more points)**
**EDUCATION**	• Skin cancer warning signs<br>• Self-exam instructions	• Skin cancer warning signs<br>• Monthly self-exam instructions	• Skin cancer warning signs<br>• Monthly self-exam instructions<br>• Medical provider skin exam	• Skin cancer warning signs<br>• Monthly self-exam instructions<br>• Medical provider skin exam
**MEDICAL PROVIDER EXAM**	See a medical provider for any suspicious lesions	• See a medical provider for any suspicious lesions<br>• Consider a yearly full body skin exam by a medical provider	• At least annual skin exam with a medical provider<br>• Consider dermatology referral for skin exam every year or whenever a suspicious lesion is found<br>• Add annual screening to health maintenance	• Refer to dermatology for full-body skin exam and continued management<br>• Add annual or biannual screening to health maintenance

Enroll Your Patients into Epic's Health Maintenance

Go to the "HM" activity in a Patient Chart to create a screening schedule for your patient, in accordance with the Melanoma Risk Score by using one of the following HM codes:

Health Maintenance Codes	
**Health Maintenance Code Title**	**HM Code**
Derm: Healthy Skin Follow Up Every 1 Year	361
Derm: Melanoma Follow Up Every 12 Months	360
Derm: Melanoma Follow Up Every 3 Months	357
Derm: Melanoma Follow Up Every 4 Months	358
Derm: Melanoma Follow Up Every 6 Months	359

The following ICD-10 codes will automatically complete the Health Maintenance codes when the diagnosis is added to the problem list, or anytime in any encounter:

ICD-10 Codes to Satisfy HM	
**Title**	**ICD-10-CM**
Screening for malignant neoplasm of the skin	Z12.83
Screening for malignant neoplasm of skin	Z12.83
Screening, malignant neoplasm, skin	Z12.83
Skin cancer screening	Z12.83
Skin exam for malignant neoplasm	Z12.83
Screening exam for skin cancer	Z12.83
Skin exam, screening for cancer	Z12.83
Screening for skin cancer	Z12.83
Encounter for screening for malignant neoplasm of skin	Z12.83

Only use these codes when you have done a full body skin exam to screen for skin cancer

Adding Patient Handouts to the After Visit Summary

While charting under “Patient Instructions”, type in the SmartPhrase ‘.SKINCANCERAVS’

If you only wish to include information on the Mole Mapper app, use the SmartPhrase ‘.MOLEMAPPER’

https://www.ohsu.edu/sites/default/files/2020-06/Skin%20Cancer%20Education%20AVS%20vFNL.pdf

What you need to know about:

Melanoma and other skin cancers

Melanoma

Melanoma is the most deadly form of skin cancer. When found early, it is almost always curable, which is why it is important to check your skin and talk to your health care provider if anything looks different.

To catch melanoma

- Check your skin every month for new or changing moles or spots.
- Let your medical provider know if you see any of the warning signs of melanoma or other skin cancers.

What does melanoma look like?

Look for new moles (spots on your skin) or moles that are changing in size, shape or color. As you get older, your moles may slowly change, but a mole should never change quickly (within a few weeks or months).

Melanoma can happen anywhere – not just areas exposed to the sun. When you are checking your skin, be sure to look at your whole body.

Melanoma warning signs

A new mole (especially if you are 55 or older) or a changing mole.

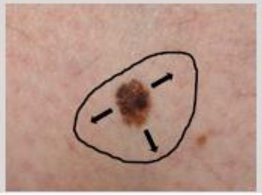

One part of the mole that is growing differently from the rest of the mole. Look for difference in color, shape or size.

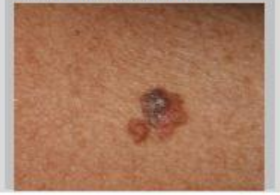

A mole that is different from your other moles

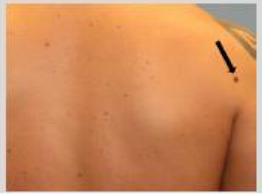

What does melanoma look like?

Below are a few examples of how melanoma might look.

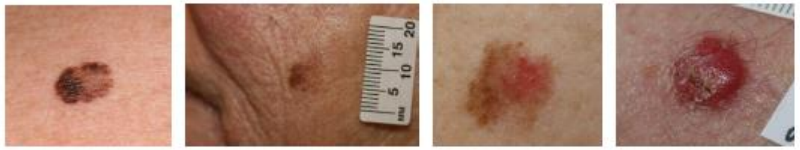

Note that just because a mole is different, does not mean it is melanoma. You should always talk to your health care provider if you see any melanoma warning signs.

Citation: Mole images: 'SkinCancer909: a textbook of skin cancer for medical students' by Jonathan Rees. www.skincancer909.com

Other types of skin cancer

Not all skin cancers are melanoma, although melanoma is the most dangerous.

The most common other types of skin cancer include:

- Squamous cell carcinoma
- Basal cell carcinoma

These skin cancers are usually found on parts of the body that get a lot of sunlight, including your:

- Face
- Ears
- Top of the head
- Neck
- Arms

Squamous Cell Carcinoma (SCC)

Squamous cell carcinomas are cancerous growths that look like scaly or crusty lumps or sores.

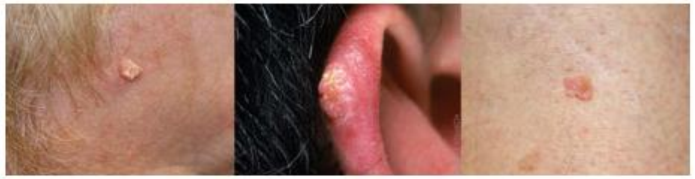

Actinic Keratosis (AK)

An actinic keratosis (AK) is a rough, scaly patch caused by sun damage. Although AKs are not cancer, they are precancerous and can become squamous cell carcinoma if they are not treated.

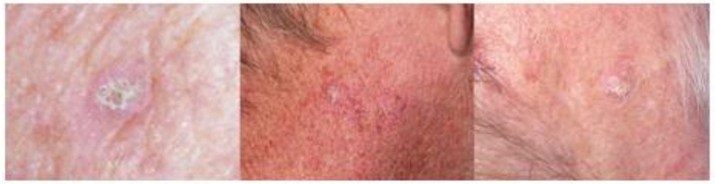

Basal Cell Carcinoma (BCC)

Basal cell carcinomas are the most common form of skin cancer. BCCs often have edges that bleed and scab. They might also look like a black, blue, brown, or a slightly scaly spot that grows over time.

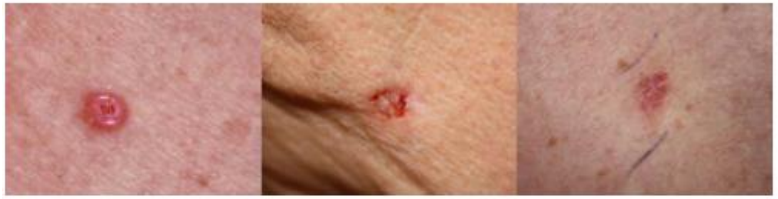

Preventing skin cancer

Sun protection

Protecting yourself from the sun is one of the best ways to prevent skin cancer. UVB rays from the sun, which cause skin cancer, are strongest from 10 am – 3 pm, so it is important to be especially careful during those times. Whenever you are in the sun:

- Wear clothes that protect your skin from sunlight (look for the UPF tag)
- Wear a wide-brimmed hat that shades your face and ears
- Wear sunglasses
- Stand in the shade whenever possible
- Apply sunscreen every two hours while out in the sun
    - Re-apply more often if you are sweating or swimming
    - Sunscreen alone is not enough to protect you from skin cancer; it should be used *in addition* to the other sun safety tips.

What sunscreen should I use?

- Find a sunscreen with an SPF of at least 30 and a label that says "broad-spectrum" protection.
- **Ingredients are important**. The best sunscreens use minerals, such as zinc oxide or titanium dioxide, instead of chemical ingredients to block the sun. They are considered safe by the FDA, even for children.
- Sunscreens that use chemicals are okay, but not ideal; we do not know the long-term safety of chemical-based sunscreens.
- Recommended brands:
    - Blue Lizard Baby
    - Banana Boat Baby
    - Trader Joe's Mineral
    - Neutrogena Pure & Free Baby
    - CoTz
    - Elta MD UV Pure Broad-Spectrum
- Remember: Any sunscreen is better than no sunscreen at all.

Self-exams

Most skin cancer is first seen by you or your partner rather than your doctor. To do a good skin check:

- Look at your whole body, including your fingernails and between your toes.
- Look at each part of your body in the same order every time to make sure you don't miss any spots.
- Use a mirror to look at the top or back of your head, back, under your breasts, genitals or buttocks.
- If you have a partner, they can help check hard-to-see areas.
- Let your health care provider know about any new or changing spots.

Free OHSU MoleMapper App (available on your iPhone or iPad)

Change or growth of moles is one of the strongest signs of skin cancer. With the MoleMapper app, you can use your iPhone or iPad to photograph and track your moles each month. The MoleMapper can help you and your health care provider see if your moles are changing in a concerning way over time.

To download: go to the App Store, search for "MoleMapper Melanoma Study," and tap "GET."

More information about preventing, finding and treating skin cancer

Visit www.StartSeeingMelanoma.com or www.WarOnMelanoma.org.

What does the "War on Melanoma™" mean?

The War on Melanoma™ is an OHSU research program designed to stop death due to melanoma through early detection. If you have any questions about the program, you can email WarOnMelanoma@ohsu.edu, call 844-300-SPOT (7768), or visit www.WarOnMelanoma.org.

Interested in research opportunities? Stay up-to-date by joining the Melanoma Community Registry: www.ohsu.edu/war-on-melanoma/melanoma-community-registry.

Questions?

If you have any questions about the app, you can contact WarOnMelanoma@ohsu.edu or call 844-300-SPOT (7768).
