# Supplementary material for: Oregon primary care providers as a frontline defense in the War on Melanoma™: improving access to melanoma education
Source: Front Med (Lausanne). 2025 Mar 14;12:1427136. doi: 10.3389/fmed.2025.1427136 (PMC11949923; doi:10.3389/fmed.2025.1427136)
Supplement: Supplementary file 4 [file Data_Sheet_4.pdf]

Skin Cancer Screening Reference Guide

Screening Recommendations

Populations at risk for developing melanoma

Adults aged 35–75 years should be screened at least annually with a total body skin examination when presenting with one or more of the following risk factors:

- Personal history of skin cancer, pre-cancerous lesions, or predisposing genetic mutation;
- Family history suggestive of a predisposition toward melanoma; or
- Physical features suggestive of susceptibility for skin cancer.

See over for a table that provides more details on risk factors.

Intake Form

Doorway risk assessment

- Fair complexion
- Blonde, red, or light brown hair
- Blue, green, or hazel eyes
- Light skin colors (Fitzpatrick I–III, Fitzpatrick 1988)
- Numerous freckles
- Many visible moles
- Evidence of sun-damaged skin

Key Clinical History Questions

- Do you have a history of skin cancer?
- Have you ever had a mole biopsied?
- Do you have a history of immunosuppression?
- Do you have a history of severe UV exposure?
- How many sunburns did you have as a child?
- How many blistering sunburns have you had in your lifetime?
- Have you ever used a tanning bed or solarium?
- Do you have a family history of skin cancer?

Diagnosis Codes

Atypical nevus *(need to specify site)* — D22.X
Family history of melanoma — Z80.8
Family history of skin cancer — Z80.8
Freckles — L81.2
History of atypical nevus — Z87.898
History of sun-damaged skin — Z87.2
Multiple pigmented nevi of the trunk and extremities — D22.7
Personal history of melanoma — Z85.820
Personal history of skin cancer — Z85.828
Personal risk factors not otherwise specified — Z91.89
Screening for skin cancer — Z12.83
Skin tanning due to UV light — L56.8
Sun-damaged skin — L57.8
Tanning bed use — Z91.89

Sample EPIC SmartPhrase (dot phrase) for Risk Factor Assessment

- Fitzpatrick skin type I–III: {YES/NO:63}
- Blond or red hair: {YES/NO:63}
- >40 total nevi:{YES/NO:63}
- ≥2 atypical nevi: {YES/NO:63}
- Many freckles: {YES/NO:63}
- Severely sun-damaged skin: {YES/NO:63}
- History of blistering or peeling sunburns: {YES/NO:63}
- History of indoor tanning: {YES/NO:63}
- Personal history of melanoma, actinic keratoses, or nonmelanoma skin cancer: {YES/NO:63}
- Personal history of CDKN2A (or other high-penetrance gene) mutation carrier: {YES/NO:63}
- Personal history of immunocompromise: {YES/NO:63}
- Family history of melanoma in one or more first degree family members: {YES/NO:63}
- Family history suggestive of a hereditary predisposition to melanoma: {YES/NO:63}

SmartPhrase Name	Purpose
.MELRISK	Melanoma risk assessment
.SKINCANCERAVS	Skin cancer education after-visit summary handout
.MELHPI	Melanoma history of present illness
.MELPE	Melanoma physical examination
.MELROS	Melanoma review of systems
.SKINCANCERPE	Skin cancer physical examination
.SKINCANCERPUNCHBX	Skin cancer single punch biopsy
.SKINCANCERPUNCHBXS	Skin cancer multiple punch biopsies
.SKINCANCERSHAVEBX	Skin cancer single shave biopsy
.SKINCANCERSHAVEBXS	Skin cancer multiple shave biopsies
.MOLEMAPPER	OHSU MoleMapper information & download instructions

RECOMMENDATIONS STRATIFIED BY MELANOMA RISK LEVEL (Add Risk Assessment Points together)				
	**Low Risk (Total: 0 points)**	**Some Risk (Total: 1-3 points)**	**Moderate Risk (Total: 4-8 points)**	**High Risk (Total: 9 or more points)**
**EDUCATION**	• Skin cancer warning signs<br>• Self-exam instructions	• Skin cancer warning signs<br>• Monthly self-exam instructions	• Skin cancer warning signs<br>• Monthly self-exam instructions<br>• Medical provider skin exam	• Skin cancer warning signs<br>• Monthly self-exam instructions<br>• Medical provider skin exam
**MEDICAL PROVIDER EXAM**	See a medical provider for any suspicious lesions	• See a medical provider for any suspicious lesions<br>• Consider a yearly full body skin exam by a medical provider	• At least annual skin exam with a medical provider<br>• Consider dermatology referral for skin exam every year or whenever a suspicious lesion is found<br>• Add annual screening to health maintenance	• Refer to dermatology for full-body skin exam and continued management<br>• Add annual or biannual screening to health maintenance

Risk level	Melanoma risk factors	Melanoma RR/OR/SIR	Reference
**Elevated risk**	1 atypical nevus *vs* 0	1.5	Gandini I 2005
	Total common nevi 16 Gandini III 2005 *vs* <15	1.5	Gandini I 2005
	Blue eye color *vs* dark	1.5	Gandini III 2005
	Hazel eye color *vs* dark	1.5	Gandini III 2005
	Green eye color *vs* dark	1.6	Gandini III 2005
	Light brown hair *vs* dark	1.6	Gandini III 2005
	Indoor tanning ever use in any gender *vs* never use	1.7	Lazovich 2010
	Fitzpatrick II *vs* IV	1.8	Gandini III 2005
	Fitzpatrick III *vs* IV	1.8	Gandini III 2005
	History of sunburn *vs* no history	2.0	Gandini II 2005
	Blond hair *vs* dark	2.0	Gandini III 2005
	2 atypical nevi *vs* 0	2.1	Gandini I 2005
	Fitzpatrick I *vs* IV	2.1	Gandini III 2005
	High density of freckles *vs* low	2.1	Gandini III 2005
	Total common nevi 41–60 *vs* <15	2.2	Gandini I 2005
**Moderately elevated risk**	Family history of melanoma in one or more first degree relative	1.7–3.0	Gandini III2005, Ford 1995, Kefford 1999
	3 atypical nevi *vs* 0	3.0	Gandini I 2005
	Total common nevi 61–80 *vs* <15	3.3	Gandini I 2005
	Red hair *vs* dark	3.6	Gandini III 2005
	CLL	3.9	Olsen and Lane 2016
	History of AK and/or KC *vs* no history	4.3	Gandini III 2005
	Indoor tanning use in women aged 30–39 years *vs* never use	4.3	Lazovich 2016
	4 atypical nevi *vs* 0	4.4	Gandini I 2005
**Marked risk**	Transplant recipient *vs* general population	2.2–4.6	Robbins 2015, Fattouh 2017
	Indoor tanning use in women aged <30 years *vs* never use	6.0	Lazovich 2016
	5 atypical nevi *vs* 0	6.4	Gandini I 2005
	Total common nevi 101–120 *vs* <15	6.9	Gandini I 2005
	Personal history of melanoma	8.2–13.4	Bradford 2010
	CDKN2A mutation carrier	14–28	Begg 2005

Reference
Johnson MM, Leachman SA, Aspinwall LG, Cranmer LD, Curiel-Lewandrowski C, Sondak VK, Stemwedel CE, Swetter SM, Vetto J, Bowles T, Dellavalle RP, Geskin LJ, Grossman D, Grossmann KF, Hawkes JE, Jeter JM, Kim CC, Kirkwood JM, Mangold AR, Wong, MK. (2017). Skin cancer screening: recommendations for data-driven screening guidelines and a review of the U.S. Preventive Services Task Force controversy. *Melanoma Management*, 2017, Vol. 4, Issue 1, pp 13–37.
