# Supplementary material for: Oregon primary care providers as a frontline defense in the War on Melanoma™: improving access to melanoma education
Source: Front Med (Lausanne). 2025 Mar 14;12:1427136. doi: 10.3389/fmed.2025.1427136 (PMC11949923; doi:10.3389/fmed.2025.1427136)
Supplement: Supplementary file 5 [file Data_Sheet_5.pdf]

Worried about health care costs?

Financial services

At many medical offices, a request for financial assistance may be made at any point before, during, or after the provision of care. Ask about the application process and qualification for financial assistance.

Questions to ask your insurance provider

If you have insurance, but you are worried about how much a visit or procedure might cost, you can call the number listed on the back of your insurance card to find out more about:

- Do you have insurance and does it pay for skin cancer screenings?
- Does your insurance pay for examinations of suspicious moles?
- Do you need a referral to see a dermatologist or do you need to see a primary care provider?
- Do you have a co-pay (an amount or percent of money you owe for covered visits)
- Do you have a deductible (the amount of money you have to meet before insurance will pay)
- Do you have a maximum (the most that insurance will pay, sometimes per year)

Understand insurance terms

The glossary at healthcare.gov provides definitions for common health insurance terms or health coverage options such as co-pay, deductible and more.

www.healthcare.gov/glossary

Visit OregonHealthCare.gov to find out about coverage options.

The Oregon Health Plan (free coverage based on income or other factors), private plans sold on HealthCare.gov, and Medicare are coverage options for people who either do not get health insurance through a job or who qualify additional coverage.

- Learn about what plan might be best and most affordable for you.
- See if you can get financial help to pay for your plan.

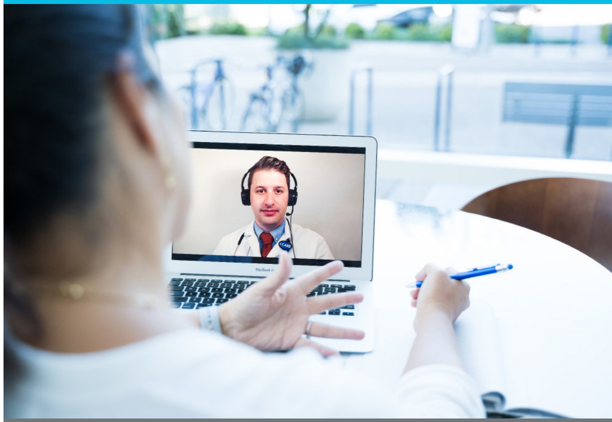

eVisits

OHSU offers virtual (online) visits. For more information visit **www.waronmelanoma.org**
