# Supplementary material for: Oregon primary care providers as a frontline defense in the War on Melanoma™: improving access to melanoma education
Source: Front Med (Lausanne). 2025 Mar 14;12:1427136. doi: 10.3389/fmed.2025.1427136 (PMC11949923; doi:10.3389/fmed.2025.1427136)
Supplement: Supplementary file 6 [file Data_Sheet_6.pdf]

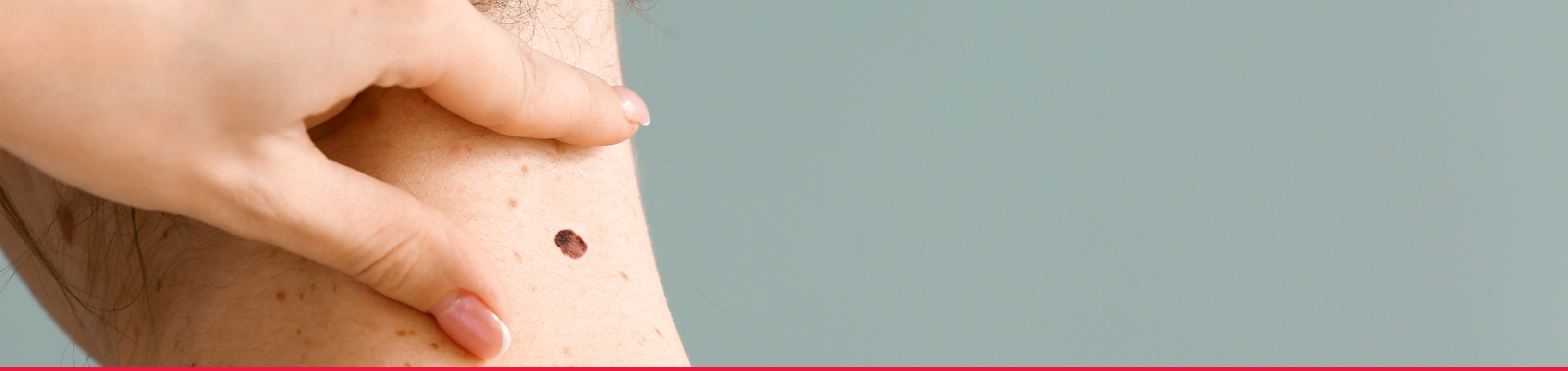

Finding **Melanomas**

Your Guide to **Self Skin Cancer Screening**

Melanomas are a type of skin cancer - most often caused by sun damage - that can spread to different parts of the body (metastasize). **Melanomas can be fatal**.

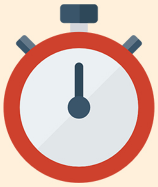

Early detection is key

99% of patients whose melanoma is caught **BEFORE** it spreads will survive at least 5 years.

30% of patients whose melanoma is caught **AFTER** it spreads will survive at least 5 years.

Examine your skin for warning signs

Look for something **different:**

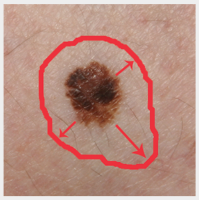

A new (especially if you are 55 or older) or **changing** mole

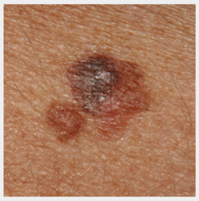

One part of the mole is growing differently than the rest (look for **difference in color, shape, size**)

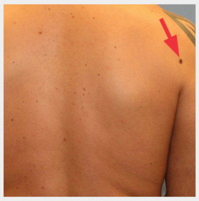

A mole **different from your other moles**, often referred to as “ugly ducklings”

Tips for self skin cancer screening

- Check your skin **once a month**
- Use **a mirror or a partner** to help you look in hard-to-see areas such as your scalp, back, genitals, soles of your feet
- Take **photos** of moles you want to track. If you have an iPhone, you can download the MoleMapper app (**www.molemapper.org**)
- **Know your risk**. Certain genetic, health and behavioral traits have been proven to elevate the risks of getting a melanoma
- If you **find something** that seems concerning, **do something** about it! Talk to your PCP, explore options for an in-person or virtual visit with a dermatologist

Learn more about melanoma early detection, take the risk assessment quiz: **www.startseeingmelanoma.com**
