# Supplementary material for: Oregon primary care providers as a frontline defense in the War on Melanoma™: improving access to melanoma education
Source: Front Med (Lausanne). 2025 Mar 14;12:1427136. doi: 10.3389/fmed.2025.1427136 (PMC11949923; doi:10.3389/fmed.2025.1427136)
Supplement: Supplementary file 7 [file Data_Sheet_7.pdf]

Did you know?

The majority of skin cancers are first detected by the **individual or a partner**, not a doctor.

While performing a self exam, it can be hard to see everywhere. Use the tips below to assure a thorough exam!

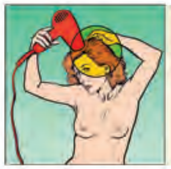

Thoroughly inspect your scalp, using a blow dryer and mirror to expose each section to view. Get a friend or family member to help if you can.

Check your hands carefully; palms and backs, between the fingers, and under fingernails. Continue up the wrists to examine both the front and backs of your forearms.

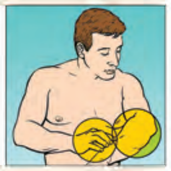

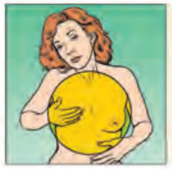

Check your neck, chest, and torso. Women should lift breasts to view the underside.

With your back to the full length mirror, use a handheld mirror to inspect the back of your neck, shoulders, and upper back.

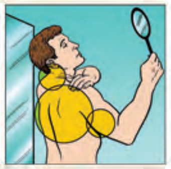

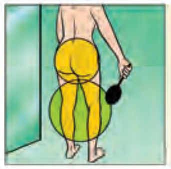

Still using both mirrors, scan your lower back, buttocks, and the backs of both legs.

Sit down; prop each leg. Use the handheld mirror to examine the genitals and mucosal area. Check the front and sides of both legs, thigh to shin; ankles, tops of feet, between toes and under toenails. Examine soles of feet and heels.

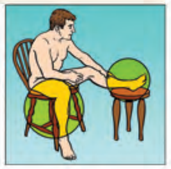

Know your risk

Research has proven that some people are at an elevated melanoma risk due to genetic, health and behavioral factors.[1] Understanding your risk level is an important tool to staying safe!

Find out your risk today!

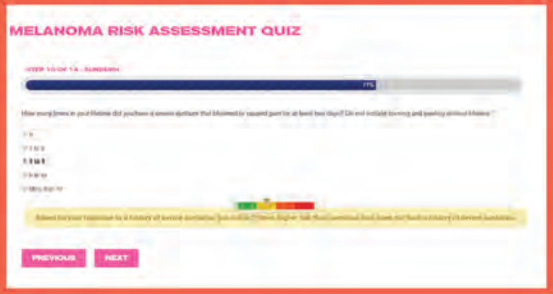

Take the quiz, and find more learning resources at:

startseeingmelanoma.com

References:
1. Johnson M, et al. Skin cancer screening: recommendations for data-driven screening guidelines and a review of the US Preventive Services Task Force controversy https://www.ncbi.nlm.nih.gov/pmc/articles/PMC5480135/ Accessed April 1, 2020.
2. SEER statistics. https://seer.cancer.gov/statfacts/html/melan.html. Accessed October 17, 2019.

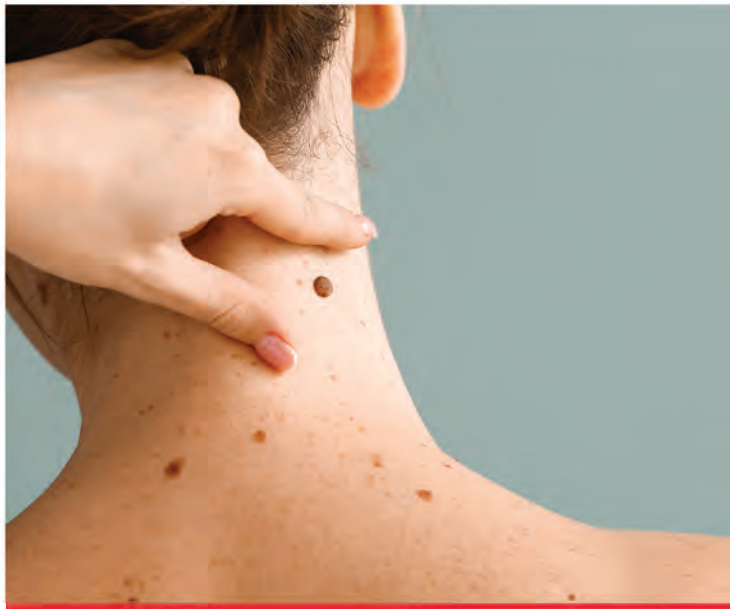

Finding **Melanomas**

Your guide to self skin cancer screening

Understanding melanoma

Melanomas are a type of skin cancer caused by sun, tanning, or other damage that can spread to different parts of the body (metastasize). Melanomas can be fatal.[1]

Melanoma cases are on the rise[2]

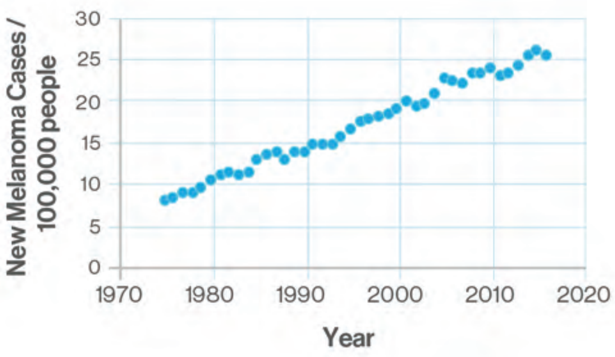

Early detection is key!

Early detection of melanoma is better.[1,2]

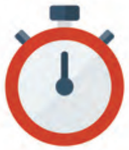

Early Stage

99% of patients whose melanoma is caught **BEFORE** it spreads will survive at least 5 years.[2]

Metastatic

30% of patients whose melanoma is caught **AFTER** it spreads will survive at least 5 years.[2]

Your doctor can help catch melanoma early through a full-body screen.[1]

Examine your skin for warning signs

You should examine your skin at least every month for melanoma warning signs.

Look for something different:

A new (especially if you are 55 or older) or **changing** mole

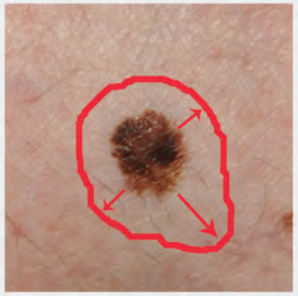

One part of the mole is growing differently than the rest (look for **difference in color, shape, size**)

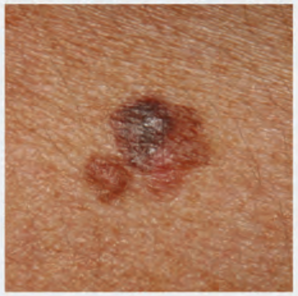

A mole **different from your other moles**, often referred to as "ugly ducklings"

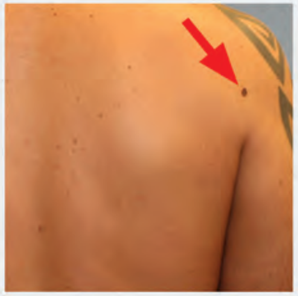

Citation: Mole images: 'SkinCancer909: a textbook of skin cancer for medical students' by Jonathan Rees. www.skincancer909.com

Melanoma can have many different appearances

Just because a mole is different, does not mean it *is* melanoma -- but you should talk to your provider if you see any warning signs. Here are a couple examples of how melanoma may look:

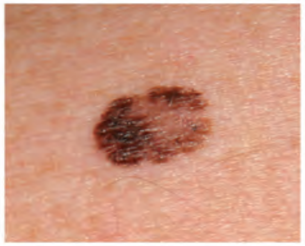
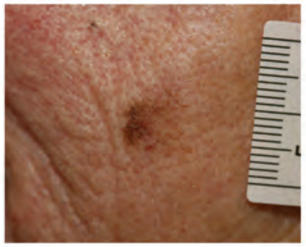
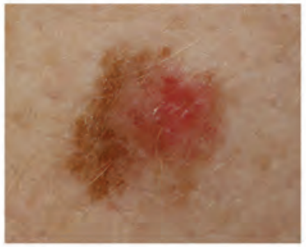
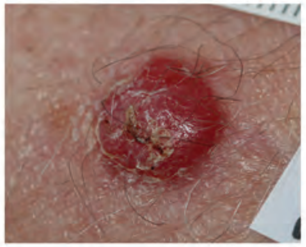

Risk factors

Some things can increase your risk of getting a melanoma, including[1]:

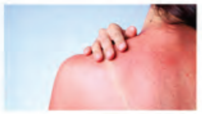
Previous severe sunburns

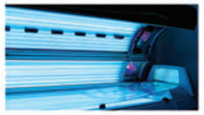
Previous use of tanning beds

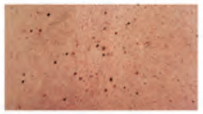
Multiple (>50) skin moles

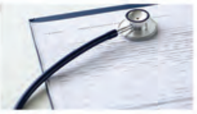
Personal history of skin cancer

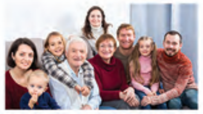
Family history of skin cancer

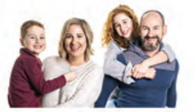
Light complexion (skin, hair, eyes)
